# French Recommendations for Sugar Intake in Adults: A Novel Approach Chosen by ANSES

**DOI:** 10.3390/nu10080989

**Published:** 2018-07-29

**Authors:** Luc Tappy, Béatrice Morio, Dalila Azzout-Marniche, Martine Champ, Mariette Gerber, Sabine Houdart, Emmanuel Mas, Salwa Rizkalla, Gérard Slama, François Mariotti, Irène Margaritis

**Affiliations:** 1 Physiology Department, Faculty of Biology and Medicine, University of Lausanne, 1005 Lausanne, Switzerland; 2 Centre Métabolique, Hôpital Intercantonal de la Broye, 1470 Estavayer-le-lac, Switzerland; 3 CarMeN laboratory, Univ-Lyon, Inserm U1060, INRA U1397, Université Claude Bernard Lyon1, INSA Lyon, BP12, F69921 Oullins CEDEX, France; beatrice.morio@inra.fr; 4 UMR Physiologie de la Nutrition et du Comportement Alimentaire, AgroParisTech, INRA, Université Paris-Saclay, 75005 Paris, France; dalila.azzout@agroparistech.fr; 5 UMR Physiologie des Adaptations Nutritionnelles, INRA, Université de Nantes, CHU Hôtel-Dieu, Place Alexis Ricordeau, 44093 Nantes CEDEX 1, France; Martine.Champ@wanadoo.fr; 6 Institut de Recherche en Cancérologie, Inserm Campus Hospitalier Val d’Aurelle, 34298 Montpellier CEDEX 5, France; mariette.gerber@sfr.fr; 7 Nutrition Risk Assessment Unit, Risk Assessment Department, French Agency for Food, Environmental and Occupational Health & Safety (ANSES), F94701 Maisons-Alfort, France; Sabine.HOUDART@anses.fr (S.H.); Irini.MARGARITIS@anses.fr (I.M.); 8 Pédiatrie-Gastro-Entérologie, Hépatologie, Nutrition et Diabétologie Pôle enfants Hôpital des Enfants 330, Avenue de Grande Bretagne-TSA 70034-31059 Toulouse CEDEX 9, France; mas.e@chu-toulouse.fr; 9 Institute of Cardiometabolism and Nutrition, ICAN, Assistance Publique Hôpitaux de Paris, Pitié-Salpêtrière Hospital, F75013 Paris, France; rizkalla_salwa@yahoo.fr; 10 Faculté de médecine René Descartes Paris V, avenue de l’école de médecine 15, F75006 Paris, France; geslama@wanadoo.fr; 11 UMR PNCA, AgroParisTech, INRA, Université Paris-Saclay, 75005 Paris, France; mariotti@agroparistech.fr

**Keywords:** dietary guidelines, cardiovascular diseases, obesity, type 2 diabetes, non-alcoholic fatty liver disease, non-communicable diseases

## Abstract

This article presents a systematic review of the scientific evidence linking sugar consumption and health in the adult population performed by a group of experts, mandated by the French Agence nationale de sécurité sanitaire de l’alimentation, de l’environnement, et du travail (ANSES). A literature search was performed by crossing search terms for overweight/obesity, diabetes/insulin resistance, dyslipidemia/cardiovascular diseases, non-alcoholic fatty liver diseases (NAFLD), and uric acid concentrations on one hand and for intake of sugars on the other. Controlled mechanistic studies, prospective cohort studies, and randomized clinical trials were extracted and assessed. A literature analysis supported links between sugar intake and both total energy intake and body weight gain, and between sugar intake and blood triglycerides independently of total energy intake. The effects of sugar on blood triglycerides were shown to be mediated by the fructose component of sucrose and were observed with an intake of fructose >50 g/day. In addition, prospective cohort studies showed associations between sugar intake and the risk of diabetes/insulin resistance, cardiovascular diseases, NAFLD, and hyperuricemia. Based on these observations, ANSES proposed to set a maximum limit to the intake of total sugars containing fructose (sucrose, glucose–fructose syrups, honey or other syrups, and natural concentrates, etc.) of 100 g/day.

## 1. Introduction

The 2004 French dietary guidelines regarding carbohydrates [1] recommended that this class of macronutrient should contribute 50 to 55% of total energy intake (TEI) and that added sugars should be limited to less than 10% of TEI. A 10% TEI for maximal sugar intake has also been advised in many national dietary recommendations, but is not actually supported by existing data, and expert reports on the health effects of sugars from the European Food Safety Agency (EFSA) 2010 [2] and the American Institute of Medicine (2002) [3] concluded that there was insufficient evidence to propose an upper limit level for this class of nutrient.

Since the turn of the millennium, an increasing number of clinical trials, prospective cohort studies, critical reviews, and meta-analyses have incriminated sugars in the pathogenesis of obesity and non-communicable diseases. This encouraged several nutrition and health agencies to revise their dietary recommendations for carbohydrates and sugars. On 20 July 2012, the French Agence nationale de sécurité sanitaire de l’alimentation, de l’environnement, et du travail (ANSES) issued an internal request to conduct the following expert appraisal: ‘‘balance of three macronutrients in daily energy intake’’. Within this expert appraisal, the authors of this article were selected as a working group (WG) to assess the scientific evidence linking sugar consumption and health in the adult population. This special report summarizes our conclusions, which were approved by the ANSES Scientific Expert Committee for human nutrition on 25 June 2015.

## 2. Materials and Methods 

### Scope of the Expertise and Strategy

A literature search was performed to assess associations between sugar consumption and the most prevalent groups of non-communicable diseases (NCDs), i.e., cardiovascular diseases, obesity, diabetes, dyslipidemia, and gout. The effects of sugars on dental caries were not addressed in the present study since this had been dealt with in the 2010 oral hygiene recommendations by the French National Authority for Health [4].

We considered carefully whether extrapolating the metabolic effects of the fructose alone to that of sugars, which are generally composed of glucose and fructose in about equivalent amounts, would be valid. We found evidence that ingestion of a protein + lipid meal + 25 g fructose alone, compared to the same protein + lipid meal containing 25 g fructose + 25 g glucose had, of course, different effects on postprandial glucose and insulin, but similar effects on postprandial plasma triglyceride and hepatic lipogenesis [5]. We also found evidence that consumption of isocaloric diets containing 35% complex carbohydrate and 25% fructose, high fructose corn syrups (HFCS), or glucose increased 24-h blood triglycerides by 4.7, 1.8, and −1.9 g/L respectively compared to a control 55% complex carbohydrate diet [6]. Another study reported that the addition of 18% total energy as sucrose, or 18% as HFCS, or 9% as fructose led to similar increases in body weight, blood triglycerides, total cholesterol, and blood pressure compared to baseline values [7]. Some studies, however, reported similar postprandial triglyceride responses after the ingestion of sucrose or glucose: fructose mixtures than after the ingestion of an isomolar amount of fructose alone [8]. We therefore estimated that extrapolating the effect of a given amount of dietary fructose to that of twice the same as fructose was approximate, but nonetheless, felt it was justified if it was the sole way to define thresholds relating sugar intake and metabolic outcomes. 

We agreed that the existence of a link would be retained if there was evidence that sugar consumption increases the incidence of diseases or recognized risk factors for diseases, i.e., for obesity, changes in energy intake (food intake) or energy expenditure (physical activity, energy efficiency) or ectopic lipid deposition in visceral fat or liver cells; for diabetes, pancreatic beta cell dysfunction or hepatic, adipose, and muscle insulin resistance; and for cardiovascular diseases, low HDL cholesterol, high LDL cholesterol, high total- and VLDL-triglyceride, high blood pressure, and hyperuricemia. 

In the context of this expertise, we defined sugars as digestible/absorbable monosaccharides (glucose, fructose and galactose) and disaccharides (sucrose, made up of one glucose linked to one fructose, lactose, made up of one glucose linked to galactose, maltose which is a dimer of glucose), or mixtures of fructose and glucose, such as high fructose corn syrups (HFCS). A literature search was performed in two databases (Medline and Scopus) by crossing search terms for overweight/obesity, diabetes/insulin resistance, dyslipidemia/cardiovascular diseases, non-alcoholic fatty liver diseases and uric acid concentrations on one hand and for intake of sugars on the other hand. When selecting search terms for sugars, we agreed upon the facts that (a) sugars’ digestion ends up with the absorption of glucose, fructose, and galactose in the blood stream, while starch’s digestion ends up with the absorption of glucose only; any specific metabolic effects of sugars are, therefore, likely to be mediated by fructose or galactose; (b) sucrose and fructose–glucose syrups contribute by far the largest part of our sugar intake, and the specific metabolic effect of these sugars is likely to be mediated by fructose; and (c) a major portion of the mechanistic literature available at the time of this expertise concerned pure fructose. We therefore considered that these studies were relevant and that their results could be extrapolated to the fructose component of other sugars. 

The literature retrieved initially was screened from article titles and abstracts by two members from the group, and only original human studies and meta-analyses of original human studies were retained. Quality was assessed based, for intervention studies, on study design, presence of an appropriate control, randomization, description of intervention allowing a quantitative assessment of sugars’ effects, and appropriate statistical analysis; and for prospective cohort studies, on methods used to assess dietary intake and appropriate adjustment for potential confounders (in particular, body weight, indexes of adiposity, and total energy intake). For meta-analyses, heterogeneity (coefficient *I*_2_) was also taken into consideration. The whole working group consensually made the final selection of articles and classified them into three subgroups according to the following criteria:

Mechanistic studies (studies dealing with underlying mechanisms) consisting of controlled short-duration studies providing information on the specific metabolic effects of sugars vs. other macronutrients, potential pathogenic mechanisms, etc. 

Prospective cohort studies (PCSs) that assessed the association between the consumption of sugars or sugar-sweetened beverages and the incidence of cardiometabolic risk factors or cardiovascular or metabolic diseases.

Randomized clinical trials (RCTs) included an intervention focusing on dietary intake of sugars or sugar-sweetened beverages and for which the clinical assessment criteria included weight, body composition, or markers of cardio-metabolic deregulation.

We defined that an association between sugar intake and a disease would be considered relevant to public health if all three of the following propositions were met (1) if mechanistic studies indicate that sugar intake affects known pathophysiological mechanisms leading to diseases, and that the addition of sugar to the diet of healthy or ill subjects leads to metabolic alterations consistent with such effects; (2) if PCSs reveal significant associations between sugar intake and the incidence/prevalence of either diseases or recognized risk factors for diseases. Since many non-communicable diseases (NCDs) are closely associated with obesity, only associations independent of adiposity were considered relevant; and (3) if RCTs involving an intervention on the intake of one or several foods containing sugar show significant effects on recognized risk factors compared to an appropriate control.

## 3. Results

### 3.1. Overweight and Obesity

#### 3.1.1. Mechanistic Studies

We first searched for evidence that dietary sugars may promote weight gain due to a fructose-induced decrease in energy expenditure. Contrasting with this hypothesis, the ingestion of fructose containing meals is associated with a higher postprandial energy expenditure compared to isocaloric glucose or starch containing meals [9,10,11,12,13,14,15,16,17]. Six studies showed that consumption of a high fructose diet over a 4-day to 12-week period did not change basal and postprandial energy expenditure [18,19,20,21,22,23] We therefore conclude that a fructose-induced decrease in energy expenditure cannot be retained as an obesity promoting effect of sugars.

We next searched for evidence that sugars may increase energy intake by impairing satiety. Few studies involving measurement of the effects of sugars on food intake were retrieved [24,25] and relied on single measurements of the effects of sugars as pre-loads on spontaneous food intake. They were, therefore, considered insufficiently accurate to reach conclusions. A small number of studies found that fructose intake is associated with lower anorexigenic and higher orexigenic hormone concentrations in the blood [8,26,27], and with changes in post-ingestive brain responses [28], but this was considered as only indirect evidence that sugars may promote food intake. We, therefore, conclude that there is insufficient scientific data to draw conclusions on the effects of sugars on satiety.

We also considered the hypothesis that sugars may alter adipose tissue distribution and specifically promote visceral fat accumulation. Few studies have addressed this issue with discordant results [29,30,31]. We, therefore, conclude that the number of high quality, statistically powered studies is too low to permit conclusions.

#### 3.1.2. PCSs

Eight cohort studies prospectively assessed the relationship between the consumption of sugar-sweetened beverages and body weight changes in adults [32,33,34,35,36,37,38,39]. All reported a statistically significant, positive association between the consumption of sugar-sweetened beverages and body weight gain. Some of them, however, did not adjust for total energy intake (TEI), and it was, therefore, impossible to know whether the association between the consumption of sugar-sweetened beverages and weight gain persisted at an equal level of total energy intake. 

One meta-analysis of 88 PCS reported that consumption of sugars in the form of sugar-sweetened beverages (SSBs) was associated with an increased total energy intake [40]. In some of the PCS included in this meta-analysis, this increase was even superior to the energy content of the SSB.

#### 3.1.3. RCTs 

Several high quality meta-analyses of sugar intervention studies were produced by other investigators and formed the basis of our assessment. They concluded that the addition of sugar to the usual free living diet of adults is associated with a significant increase in body weight, and two of them concluded that subtraction of sugar leads to a decrease in body weight [41,42,43]. One of these meta-analyses also analysed 11 studies in which sugar isocalorically replaced other dietary carbohydrates and concluded that this intervention did not change body weight [43]. 

#### 3.1.4. Conclusions 

Based on the reviewed literature we conclude that (1) sugars do not decrease thermogenesis and basal metabolism; (2) studies that have evaluated the effects of sugars on satiety have yielded contradictory results, and do not allow to reach conclusions at this time; (3) sugar supplementation, with dietary intake otherwise left ad-libitum, is associated with body weight gain; and (4) epidemiological studies have consistently shown an association between sugar consumption and body weight gain, and between sugar consumption in the form of sugar-sweetened beverages (SSBs) and total energy intake. We conclude that there is an association between dietary sugars and body weight gain, and that it is likely to be explained by excess energy consumption associated with sugar intake. 

### 3.2. Diabetes, Glucose Homeostasis, and Insulin Sensitivity 

#### 3.2.1. Mechanistic Studies

To assess whether dietary sugar intake may be a causal factor for the development of diabetes and impaired glucose tolerance, the working group relied on mechanistic short-term controlled studies in which hepatic and/or whole-body (mainly muscle) insulin resistance were studied. The group identified nine intervention studies in which healthy lean, overweight, or obese subjects received a fructose or sucrose supplement over periods ranging from 7 days to 3 months and had their insulin sensitivity measured by euglycaemic-hyperinsulinaemic clamps [19,21,44,45,46,47,48,49,50]. Fructose supplementation did not decrease whole-body insulin-mediated glucose transport, corresponding mainly to muscle insulin sensitivity [19,21,44,45,46,47,49,50] with the exception of one study performed in middle-aged subjects with metabolic syndrome [48]. In contrast, fructose significantly decreased hepatic insulin sensitivity in four [21,46,47,51] out of five studies that incorporated this measurement [19,21,46,47,51]. This effect was observed with a high daily fructose intake (>80 g/day). 

#### 3.2.2. PCSs

One PCS showed an association between sugar intake and markers of insulin resistance [52] and two PCSs showed an association with the incidence of diabetes [53,54]. These associations were markedly attenuated when data were adjusted for body weight, however. 

#### 3.2.3. RCTs

Three meta-analyses concluded that substituting fructose for sucrose or starch significantly decreased post-prandial or day-long blood glucose and glycated haemoglobin concentrations in healthy subjects and in subjects with type 2 diabetes [55,56,57]. This was found with low daily fructose amounts, inferior to 90 g/day. 

#### 3.2.4. Conclusions

Based on the reviewed literature, we found that high fructose intake (>80 g/day) is associated with hepatic insulin resistance without hyperglycemia but is not associated with impaired insulin-mediated glucose disposal (which is a hallmark of type 2 diabetes mellitus) independently of changes in body weight. Consequently, we conclude that there was no evidence for a link between sugar intake and diabetes or impaired glucose homeostasis. In this context, the association between sugar intake and incidence of diabetes reported in cohort studies may be mediated by body weight gain. The very long-term effects of sugars on insulin sensitivity, insulin secretion, and glucose homeostasis remain unknown, however.

### 3.3. Blood Lipids and Cardiovascular Diseases

The literature search did not provide strong evidence for a link between sugar consumption and LDL cholesterol or HDL cholesterol. There was, however, a link between sugar consumption and fasting and post-prandial triglyceride concentrations, which are recognized as independent predictors of cardiovascular diseases [58,59].

#### 3.3.1. Mechanistic Studies

After reviewing the scientific literature pertaining to mechanisms responsible for a fructose-induced increase in blood triglyceride concentration, the WG observed that no single unequivocal mechanism had been identified. Fructose has long been known to stimulate de novo lipogenesis [60], but this effect is shared with glucose [61] and maltodextrins [62]. That stimulation of hepatic de novo lipogenesis contributes to the development of hypertriglyceridemia has been suggested by mechanistic studies showing that ^13^C carbons from ingested ^13^C-labelled fructose can be recovered in glycerol and fatty acids of blood triglycerides immediately after a meal [63]. In addition, it has been observed that fructose may decrease postprandial triglyceride-rich lipoprotein clearance; a lower postprandial insulin response with fructose compared to starch or glucose may contribute to this effect through lower stimulation of adipose lipoprotein lipase [26,63]. Additional effects of fructose on triglyceride-rich lipoprotein kinetics cannot be ruled out. 

#### 3.3.2. PCSs

Two prospective cohort studies [35,64] reported an association between sugar-sweetened beverage consumption and the development of hypertriglyceridemia in the general adult population. The data available from these studies, however, do not allow this effect to be attributed unequivocally to sugar intake independently of total energy intake. 

#### 3.3.3. RCTs

Fourteen RCTs were identified during the period covered by the WG assessment. These studies compared blood lipid concentrations after a fructose-containing sugar supplementation (fructose, fructose + glucose, sucrose or high fructose corn syrup) to pre-supplementation values, or to supplementation with glucose or fat. All studies reported an increase in fasting or postprandial blood triglyceride with supplementation [6,8,19,21,26,27,29,30,31,65,66,67]. In addition, one study reported increased blood LDL cholesterol and apoB [6]. Two meta-analyses of RCTs published before 2006 and 2009, respectively, also reported that fructose intake is associated with increases in fasting and postprandial blood triglyceride in healthy subjects [57] and subjects with type 2 diabetes [57,68]. These two meta-analyses furthermore identified that postprandial blood triglyceride concentrations increase with daily fructose intakes of 50–60 g or above. One of them [57] also found that fasting blood triglyceride increases with daily fructose intake of 100 g or above. 

#### 3.3.4. Conclusions

Based on the reviewed literature summarized above, we conclude that there is no association between sugar intake and LDL or HDL cholesterol. In contrast, there is an association between sugar intake and fasting and postprandial blood triglyceride concentration, which are independent cardiovascular risk factors. The minimal daily fructose dose associated with increased postprandial triglyceride concentration is estimated to be 50 g/day, corresponding to a fructose content of 100 g of sugar.

### 3.4. Intrahepatic Lipids and Non-Alcoholic Fatty Liver Disease

Non-Alcoholic Fatty Liver Disease (NAFLD) is highly prevalent in affluent countries and shows a close association with insulin resistance and cardiometabolic risk factors. Several authors have suggested that this disease may be primarily due to excess dietary sugar intake.

#### 3.4.1. Mechanistic Studies

Several short-term supplementation studies have shown that the addition of fructose to the diet significantly increases intrahepatic fat concentrations [21,22,29,31,69,70]. These studies involved the addition of large amounts of fructose to weight-maintenance diets. Two studies, however, did not observe any significant effect with about 100 g or 150 g fructose per day for 4 weeks. [19,30]. One study observed that hypercaloric diets containing either 30% fructose or 30% glucose caused similar increases in intrahepatic fat concentration compared to a weight-maintenance low-sugar diet. Another observed that isocaloric substitution of 30% starch with either fructose or glucose in a weight-maintenance diet did not change intrahepatic fat concentration significantly [71]. This effect may, therefore, be related to excess energy intake or excess carbohydrate intake rather than to fructose per se.

#### 3.4.2. PCSs 

We did not retrieve any PCSs that had assessed the association between sugar consumption and NAFLD during the ANSES assessment period. One case-control study reported that sugar consumption was higher in a group of patients with NAFLD than in unaffected controls [72]. One cross-sectional study of 2003 subjects reported an inverse correlation between fructose consumption and a score of intrahepatic fat content based on blood clinical chemistry [73]. One retrospective study [74] indicated that, among 427 patients with non-alcoholic fatty liver disease, fructose intake was significantly associated with the degree and severity of hepatic fibrosis. 

#### 3.4.3. Conclusions

Based on the reviewed literature summarized above, we conclude that a very high fructose intake can increase the intrahepatic fat concentration, but that the scientific data available at the time of assessment is, to date, not sufficient to assess the association between sugar intake and NAFLD. We nonetheless acknowledge that fructose or sucrose can induce oxidative stress and trigger the formation of aldehydes in the liver of rodents [75,76], and that these effects, if present in humans, may favor liver inflammation and promote the progression of a hepatic steatosis to a steatohepatitis. 

### 3.5. Blood Uric Acid Concentration

#### 3.5.1. Mechanistic Studies

Rodent studies have indicated that fructose-induced increases in uric acid concentration impair endothelial cell function and cause “vascular” insulin resistance [77]. One study performed in overweight middle-aged men further suggested that the same effects are present in humans [78]. 

#### 3.5.2. PCSs 

Two prospective cohort studies, one on 46,393 North American men [79] and one on 78,906 North American women [80] reported that the consumption of sugar-sweetened beverages is associated with the development of gout. This association remained significant after adjustment for body weight.

#### 3.5.3. RCTs 

We identified four intervention studies involving dietary supplementation with fructose [21,22,30,81]. Three of them reported an increase in blood uric acid after fructose [21,22,81]. Two studies included a comparison between a high fructose diet and a high glucose diet. One (81) reported a higher increase in blood uric acid concentration with fructose than with glucose, whereas the other [22] reported similar effects with fructose and glucose. One meta-analysis [82] of 21 RCTs with fructose intake for more than 7 days concluded that the uric acid concentration did not change when fructose isocalorically replaced other dietary carbohydrates (18 studies), but increased significantly when high amounts of fructose (213–219 g/day) were added to a weight-maintenance diet (three studies).

#### 3.5.4. Conclusions

The reviewed literature provided somewhat conflicting information. On one hand, mechanistic studies and RCTs indicate that sugar interventions are not associated with changes in uric acid concentration, unless they involve very large amounts of fructose together with excess total energy intake. Even under such extreme conditions, the increase in blood uric acid concentration is of small magnitude, and long-term consequences on health remain unknown. On the other hand, the consumption of sugar-sweetened beverages (SSBs) is associated with the development of gout in PCSs. We, therefore, conclude that there is currently insufficient information to conclude an association between sugar intake and blood uric acid concentration.

### 3.6. Recommendations Issued in the Final ANSES Report 

Based on the review of the literature (summarized in Table S1), we conclude that there is strong evidence that sugar intake is associated with body weight gain and with the development of hypertriglyceridemia, hepatic insulin resistance, hyperuricemia, and with an increased intrahepatic fat content. We also found indirect evidence that it may be associated with increased risks of diabetes and cardiovascular diseases. It was not possible to accurately identify the level of intake above which sugars exert deleterious effects on diseases-related outcomes. We nevertheless considered it necessary to propose a maximum limit to sugar intake. Our literature review indicates that the lowest daily fructose intakes with significant documented effects are 50 g/day for blood triglycerides, 80 g/day for hepatic insulin resistance, >150 g/day for intrahepatic fat, and >200 g/day for uric acid. In addition, for intrahepatic fat and uric acid, these thresholds were valid only in trials in which high fructose intake was associated with energy intake above requirement. We, therefore, selected a daily intake of 100 g sugar as an upper limit, assuming that fructose constitutes close to 50% of most commonly consumed sugars.

### 3.7. Major Advances in the Field Since the End of the Assessment (May 2015)

Many publications on the health effects of sugars were published during the period elapsed between the end of the current assessment and the time that this summary was written. Here, we review all meta-analyses addressing sugar intake and health outcomes in the general population and published between May 2015 and December 2017 in order to assess whether some of our conclusions should be revised based on novel findings. Individual studies (included in these meta-analyses or not) were, however, not reviewed.

One meta-analysis of eleven PCSs concluded that the risk of obesity is significantly higher in subjects consuming sugar-sweetened sodas than in those who do not consume sodas. Three of these studies also observed that the consumption of artificially-sweetened sodas is also associated with an increased risk for obesity [83]. One meta-analysis of eight RCTs reported an increased risk of hypertension in high vs. low SSB consumers; this association was significant for both males and females [84]. Two other meta-analyses, each including six PCSs, observed a dose-dependent increase in blood pressure with increasing SSB consumption [85,86]. Similarly, an association between SSB intake and the risk of coronary and cardiovascular diseases was mentioned in other meta-analyses [86,87,88]. In most of these meta-analyses, the association was attenuated, but remained significant, after adjustment for adiposity indices. One meta-analysis of 17 PCSs reported an association between SSB consumption and the risk of type 2 diabetes independently of adiposity [89]. Another meta-analysis of eight PCSs [90] specifically assessed the association between fruit juice consumption and the risk of type 2 diabetes. It reported that the consumption of fruit juices with added sugar, but not 100% fruit juice, was associated with a risk of diabetes. The association between sugar-sweetened beverages and the risk of developing NAFLD was reported in one meta-analysis of seven PCSs [91]. The relative risk of NAFLD was significantly increased with SSBs, even after adjustment for body weight and other confounding factors. The effect of adding fructose on blood lipids was reported in a meta-analysis of 51 isocaloric fructose replacement and eight hypercaloric fructose addition studies. It concluded that hypercaloric fructose increases blood triglyceride and apoB100 concentrations, while isocaloric fructose does not alter blood lipid parameters [92]. The relationship between fructose consumption and the incidence of group was assessed in a meta-analysis of two PCSs. It was concluded that fructose consumption is associated with an increased risk of developing gout, but the relationship between fructose intake and uric acid concentration blood uric acid concentrations was not assessed [93]. 

We, therefore, did not identify novel meta-analysis that contradicted our initial conclusions. In line with the data available at the time we performed our assessment, these recent publications further document that the association between sugar and risk of cardiovascular and metabolic diseases is, at least in part, independent of sugar-induced changes in body weight. Most of them specifically addressed SSBs and cannot be extrapolated to total sugar consumption. In addition, associations may be explained, in part, by confounding factors, such as eating habits or other uncontrolled lifestyle variables.

## 4. Discussion

Similar assessments of the health effects of dietary sugars have been made in parallel by several other regional, national, or international agencies, which released their conclusions almost simultaneously. The World Health Organisation ( WHO) report [94] recommended that free sugar intake should represent less than 10% total energy for children and adults (corresponding to about 50 g/day for a woman with a daily energy requirement of 2000 kcal/day and about 60 g/day for a man with a daily energy requirement of 2500 kcal/day). The report made an additional, conditional recommendation to lower it further to 5%. The UK SACN report [95] recommended that free sugar intake should be less than 5% of total energy for age groups >2 years. The recent US Department of Agriculture dietary guidelines for Americans recommended that added sugar intake should not exceed 10% of total energy intake [96]. 

Although all reports were drawn up on the basis of very similar literature research, they differ somewhat in the way information was handled. WHO and UK Scientific Advisory Committee on Nutrition (SACN) recommendations were essentially based on prospective cohort studies and randomized clinical trials, and hence, were based on clinical and epidemiological information. This approach has the advantage of resting on data from very large samples of representative subjects of the general population. However, it has the major weakness that prospective cohort studies rely on unreliable quantitative assessment of food intake, and hence, may yield erroneous estimates of dose–response relationships. Our approach put more emphasis on mechanistic studies and RCTs to assess thresholds of intake above which sugar intake may negatively impact NCD risk factors. An early step of this procedure was to postulate that the specific effects of sugars are attributed to their fructose component and to rely on studies having assessed the effects of pure fructose to obtain reliable such thresholds. One potential bias of this approach is that it assumes that the effect of 50 g of fructose can be extrapolated to the effect of 100 g sucrose. 

Another major difference was that we assessed the effects of total sugars rather than “added sugars” (USA) or “free” sugars (WHO, SACN). The definition of “added” or “free” sugars remains debated, and can show considerable variation, however [97]. Indeed, in many studies, the description of “added” is insufficiently accurate for analysis. Of course, there is strong evidence that fruit and vegetable consumption is associated with beneficial effects on NCD risk. The available data are, however, insufficient for assessing the specific effects of sugars contained in fruits and vegetables on the total sugar-induced metabolic and health effects, since the beneficial effects of these foods may be linked to other factors (micronutrients, dietary fibers, feeding pattern, or low caloric density). Concerning lactose and galactose, which are the main other sugars naturally present in foods and consumed by the general population, systematic assessment of their health effects is not available. However, there are no data suggesting that they are deleterious to health at the current level of consumption observed in the population. Based on these considerations, the recommendation has been established on total fructose-containing sugars. 

Accordingly, the new French recommendations set an upper limit of 100 g total sugar/day but reiterate that fruit and vegetable intakes are to be promoted [98]. The optimisation tool deployed by ANSES to update food consumption guidelines for the French population (ANSES, 2017) shows that when these two constraints are observed simultaneously (with 41 dietary reference values and toxicological reference values for around a hundred contaminants) fruits provide around 40 g of sugars, and vegetables provide 6 g of sugars. This result is consistent with a daily amount of free or added sugars in the 5–10% TEI range, and thus fits with the WHO and SACN recommendations.

## 5. Conclusions

In light of the assessment carried out by the WG, the ANSES Expert Committee (CES) on “Human Nutrition” concluded that there is a clear association between sugar intake and weight gain. The effect of sugars is mediated by the excess energy intake. Moreover, the CES concluded that there is a clear association between the intakes of dietary sugars and blood lipids. Concerning other health effects, there is no strong scientific evidence of a direct association between sugar consumption and diabetes, NAFLD, or CVD. However, epidemiological studies and plausible pathophysiological mechanisms raise concerns that sugar consumption may have adverse health effects. This has mainly been documented for SSBs. Whether sugar-sweetened drinks are less satiating per se than solid sugars and thereby, subsequently contribute to increases in energy intake through a reduced dietary energy compensation needs to be documented more thoroughly by mechanistic studies.

On the basis of presently available data, it is not possible to distinguish the health effects of sugars naturally present in food from those of added sugars. In this context, the CES recommends that total daily sugar intake (including five portions of fruits and vegetable but excluding lactose and galactose from dairy products) should not exceed 100 g/day. It should be noted that this value has only been set here for adult subjects. Additional recommendations should be established specifically for the vulnerable population of children and adolescents. From a public health policy perspective, compliance with this threshold value of 100 g/day requires effective measures aimed at reducing the consumption of added sugars. These actions should concern a wide range of spheres, from consumer education and information to regulatory measures, and these actions should be carried out in synergy. In the meantime, the research community should pursue its efforts to clarify the nature of the relationship between sugar consumption and the associated pathologies, especially in vulnerable populations, such as children and adolescents, and to explore fields that are less documented, such as cognitive psychology or neurophysiology.

## Supplementary Materials

The following are available online at http://www.mdpi.com/2072-6643/10/8/989/s1, Table S1: Key metabolic effects of sugars retained from the literature analysis.

## References

[1] AFSSA Glucides et santé: état des lieux, évaluation et Recommandations (Oct 2004). https://www.anses.fr/fr/system/files/NUT-Ra-Glucides.pdf.

[2] Agostoni C., Bresson J.-L., Fairweather-Tait S., Flynn A., Golly I., Korhonen H., Lagiou P., Løvik M., Marchelli R., Martin A. (2010). Scientific Opinion on Dietary Reference Values for carbohydrates and dietary fibre. EFSA J..

[3] Medicine Io (2005). Dietary References Intakes for Energy, Carbohydrate, Fiber, Fat, Fatty Acids, Cholesterol, Protein and Amino Acids.

[4] Haute Autorité de Santé (HAS). https://www.has-sante.fr/portail/upload/docs/application/pdf/2010-10/corriges_synthese_carie_dentaire_version_postcollege-10sept2010.pdf.

[5] Theytaz F., de Giorgi S., Hodson L., Stefanoni N., Rey V., Schneiter P., Giusti V., Tappy L. (2014). Metabolic fate of fructose ingested with and without glucose in a mixed meal. Nutrients.

[6] Stanhope K.L., Bremer A.A., Medici V., Nakajima K., Ito Y., Nakano T., Chen G., Fong T.H., Lee V., Menorca R.I. (2011). Consumption of fructose and high fructose corn syrup increase postprandial triglycerides, LDL-cholesterol, and apolipoprotein-B in young men and women. JCEM.

[7] Angelopoulos T.J., Lowndes J., Sinnett S., Rippe J.M. (2016). Fructose Containing Sugars at Normal Levels of Consumption Do Not Effect Adversely Components of the Metabolic Syndrome and Risk Factors for Cardiovascular Disease. Nutrients.

[8] Stanhope K.L., Griffen S.C., Bair B.R., Swarbrick M.M., Keim N.L., Havel P.J. (2008). Twenty-four-hour endocrine and metabolic profiles following consumption of high-fructose corn syrup-, sucrose-, fructose-, and glucose-sweetened beverages with meals. Am J. Clin. Nutr..

[9] Sharief N.N., Macdonald I. (1982). Differences in dietary-induced thermogenesis with various carbohydrates in normal and overweight men. Am J. Clin. Nutr..

[10] Tappy L., Randin J.P., Felber J.P., Chiolero R., Simonson D.C., Jequier E., DeFronzo R.A. (1986). Comparison of thermogenic effect of fructose and glucose in normal humans. Am. J. Physiol..

[11] Simonson D.C., Tappy L., Jequier E., Felber J.P., DeFronzo R.A. (1988). Normalization of carbohydrate-induced thermogenesis by fructose in insulin-resistant states. Am J. Clin. Physiol..

[12] Schwarz J.M., Schutz Y., Froidevaux F., Acheson K.J., Jeanpretre N., Schneider H., Felber J.P., Jéquier E. (1989). Thermogenesis in men and women induced by fructose vs glucose added to a meal. Am J. Clin. Nutr..

[13] Schwarz J.M., Schutz Y., Piolino V., Schneider H., Felber J.P., Jequier E. (1992). Thermogenesis in obese women: Effect of fructose vs. glucose added to a meal. AJP.

[14] Martines D., Martines V., Pasini M., Cocco G., Lora L., Varnier M., Venier G.B., Sammartano G., Naccarato R. (1994). Carbohydrate-induced thermogenesis in liver cirrhosis: Glucose vs. fructose. Nutrition.

[15] Fukagawa N.K., Veirs H., Langeloh G. (1995). Acute effects of fructose and glucose ingestion with and without caffeine in young and old humans. Metab. Clin. Exp..

[16] Blaak E.E., Saris W.H. (1996). Postprandial thermogenesis and substrate utilization after ingestion of different dietary carbohydrates. Metabo. Clin. Exp..

[17] Van Gaal L., Mertens I., Vansant G., De Leeuw I. (1999). Carbohydrate-induced thermogenesis in obese women. Effect of insulin and catecholamines. J. Endocrinol. Investig..

[18] McDevitt R.M., Poppitt S.D., Murgatroyd P.R., Prentice A.M. (2000). Macronutrient disposal during controlled overfeeding with glucose, fructose, sucrose, or fat in lean and obese women. Am. J. Clin. Nutr..

[19] Le K.A., Faeh D., Stettler R., Ith M., Kreis R., Vermathen P., Boesch C., Ravussin E., Tappy L. (2006). A 4-wk high-fructose diet alters lipid metabolism without affecting insulin sensitivity or ectopic lipids in healthy humans. Am. J. Clin. Nutr..

[20] Abdel-Sayed A., Binnert C., Le K.A., Bortolotti M., Schneiter P., Tappy L. (2008). A high-fructose diet impairs basal and stress-mediated lipid metabolism in healthy male subjects. Br. J. Nutr..

[21] Le K.A., Ith M., Kreis R., Faeh D., Bortolotti M., Tran C., Boesch C., Tappy L. (2009). Fructose overconsumption causes dyslipidemia and ectopic lipid deposition in healthy subjects with and without a family history of type 2 diabetes. Am. J. Clin. Nutr..

[22] Ngo Sock E.T., Le K.A., Ith M., Kreis R., Boesch C., Tappy L. (2010). Effects of a short-term overfeeding with fructose or glucose in healthy young males. Br. J. Nutr..

[23] Cox C.L., Stanhope K.L., Schwarz J.M., Graham J.L., Hatcher B., Griffen S.C., Bremer A.A., Berglund L., McGahan J.P., Havel P.J. (2012). Consumption of fructose-sweetened beverages for 10 weeks reduces net fat oxidation and energy expenditure in overweight/obese men and women. Eur. J. Clin. Nutr..

[24] Rodin J., Reed D., Jamner L. (1988). Metabolic effects of fructose and glucose: Implications for food intake. Am. J. Clin. Nutr..

[25] Rodin J. (1990). Comparative effects of fructose, aspartame, glucose, and water preloads on calorie and macronutrient intake. Am. J. Clin. Nutr..

[26] Teff K.L., Elliott S.S., Tschop M., Kieffer T.J., Rader D., Heiman M., Townsend R.R., Keim N.L., D’Alessio D., Havel P.J. (2004). Dietary fructose reduces circulating insulin and leptin, attenuates postprandial suppression of ghrelin, and increases triglycerides in women. J. Clin. Endocrinol. Metab..

[27] Teff K.L., Grudziak J., Townsend R.R., Dunn T.N., Grant R.W., Adams S.H., Keim N.L., Cummings B.P., Stanhope K.L., Havel P.J. (2009). Endocrine and metabolic effects of consuming fructose- and glucose-sweetened beverages with meals in obese men and women: Influence of insulin resistance on plasma triglyceride responses. J. Clin. Endocrinol. Metab..

[28] Page K.A., Chan O., Arora J., Belfort-Deaguiar R., Dzuira J., Roehmholdt B., Cline G.W., Naik S., Sinha R., Constable R.T. (2013). Effects of fructose vs glucose on regional cerebral blood flow in brain regions involved with appetite and reward pathways. JAMA.

[29] Maersk M., Belza A., Stødkilde-Jørgensen H., Ringgaard S., Chabanova E., Thomsen H., Pedersen S.B., Astrup A., Richelsen B. (2012). Sucrose-sweetened beverages increase fat storage in the liver, muscle, and visceral fat depot: A 6-mo randomized intervention study. Am. J. Clin. Nutr..

[30] Silbernagel G., Machann J., Unmuth S., Schick F., Stefan N., Häring H.U., Fritsche A. (2011). Effects of 4-week very-high-fructose/glucose diets on insulin sensitivity, visceral fat and intrahepatic lipids: An exploratory trial. Br. J. Nutr..

[31] Stanhope K.L., Schwarz J.M., Keim N.L., Griffen S.C., Bremer A.A., Graham J.L., Hatcher B., Cox C.L., Dyachenko A., Zhang W. (2009). Consuming fructose-sweetened, not glucose-sweetened, beverages increases visceral adiposity and lipids and decreases insulin sensitivity in overweight/obese humans. J. Clin. Investig..

[32] Gibbs B.B., Kinzel L.S., Gabriel K.P., Chang Y.F., Kuller L.H. (2012). Short- and long-term eating habit modification predicts weight change in overweight, postmenopausal women: Results from the woman study. J. Acad. Nutr. Diet..

[33] Bes-Rastrollo M., Sanchez-Villegas A., Gomez-Gracia E., Martinez J.A., Pajares R.M., Martinez-Gonzalez M.A. (2006). Predictors of weight gain in a Mediterranean cohort: The Seguimiento Universidad de Navarra Study 1. Am. J. Clin. Nutr..

[34] Chen L., Appel L.J., Loria C., Lin P.H., Champagne C.M., Elmer P.J., Ard J.D., Mitchell D., Batch B.C., Svetkey L.P. (2009). Reduction in consumption of sugar-sweetened beverages is associated with weight loss: The PREMIER trial. Am. J. Clin. Nutr..

[35] De Koning L., Malik V.S., Kellogg M.D., Rimm E.B., Willett W.C., Hu F.B. (2012). Sweetened beverage consumption, incident coronary heart disease, and biomarkers of risk in men. Circulation.

[36] Mozaffarian D., Hao T., Rimm E.B., Willett W.C., Hu F.B. (2011). Changes in diet and lifestyle and long-term weight gain in women and men. N. Engl. J. Med..

[37] Palmer J.R., Boggs D.A., Krishnan S., Hu F.B., Singer M., Rosenberg L. (2008). Sugar-sweetened beverages and incidence of type 2 diabetes mellitus in African American women. Arch. Intern. Med..

[38] Pan A., Malik V.S., Hao T., Willett W.C., Mozaffarian D., Hu F.B. (2013). Changes in water and beverage intake and long-term weight changes: Results from three prospective cohort studies. Int. J. Obes..

[39] Stookey J.D., Constant F., Popkin B.M., Gardner C.D. (2008). Drinking water is associated with weight loss in overweight dieting women independent of diet and activity. Obesity.

[40] Vartanian L.R., Schwartz M.B., Brownell K.D. (2007). Effects of soft drink consumption on nutrition and health: a systematic review and meta-analysis. Am. J. Public Health.

[41] Malik V.S., Pan A., Willett W.C., Hu F.B. (2013). Sugar-sweetened beverages and weight gain in children and adults: A systematic review and meta-analysis. Am. J. Clin. Nutr..

[42] Mattes R.D., Shikany J.M., Kaiser K.A., Allison D.B. (2011). Nutritively sweetened beverage consumption and body weight: A systematic review and meta-analysis of randomized experiments. Obes. Rev..

[43] Te Morenga L., Mallard S., Mann J. (2012). Dietary sugars and body weight: Systematic review and meta-analyses of randomised controlled trials and cohort studies. BMJ.

[44] Aeberli I., Hochuli M., Gerber P.A., Sze L., Murer S.B., Tappy L., Spinas G.A., Berneis K. (2013). Moderate amounts of fructose consumption impair insulin sensitivity in healthy young men: A randomized controlled trial. Diabetes Care.

[45] Black R.N., Spence M., McMahon R.O., Cuskelly G.J., Ennis C.N., McCance D.R., Young I.S., Bell P.M., Hunter S.J. (2006). Effect of eucaloric high- and low-sucrose diets with identical macronutrient profile on insulin resistance and vascular risk: A randomized controlled trial. Diabetes.

[46] Couchepin C., Le K.A., Bortolotti M., da Encarnacao J.A., Oboni J.B., Tran C., Tran C., Schneiter P., Tappy L. (2008). Markedly blunted metabolic effects of fructose in healthy young female subjects compared with male subjects. Diabetes Care.

[47] Faeh D., Minehira K., Schwarz J.M., Periasamy R., Park S., Tappy L. (2005). Effect of fructose overfeeding and fish oil administration on hepatic de novo lipogenesis and insulin sensitivity in healthy men. Diabetes..

[48] Hokayem M., Blond E., Vidal H., Lambert K., Meugnier E., Feillet-Coudray C., Coudray C., Pesenti S., Luyton C., Lambert-Porcheron S. (2013). Grape polyphenols prevent fructose-induced oxidative stress and insulin resistance in first-degree relatives of type 2 diabetic patients. Diabetes Care.

[49] Lewis A.S., McCourt H.J., Ennis C.N., Bell P.M., Courtney C.H., McKinley M.C., Young I.S., Hunter S.J. (2013). Comparison of 5% versus 15% sucrose intakes as part of a eucaloric diet in overweight and obese subjects: Effects on insulin sensitivity, glucose metabolism, vascular compliance, body composition and lipid profile. A randomised controlled trial. Metabolism.

[50] Thorburn A.W., Crapo P.A., Griver K., Wallace P., Henry R.R. (1990). Long-term effects of dietary fructose on carbohydrate metabolism in non-insulin-dependent diabetes mellitus. Metabolism.

[51] Aeberli I., Gerber P.A., Hochuli M., Kohler S., Haile S.R., Gouni-Berthold I., Berthold H.K., Spinas G.A., Berneis K. (2011). Low to moderate sugar-sweetened beverage consumption impairs glucose and lipid metabolism and promotes inflammation in healthy young men: A randomized controlled trial. Am. J. Clin. Nutr..

[52] Kelishadi R., Mansourian M., Heidari-Beni M. (2014). Association of fructose consumption and components of metabolic syndrome in human studies: A systematic review and meta-analysis. Nutrition.

[53] Fagherazzi G., Vilier A., Saes Sartorelli D., Lajous M., Balkau B., Clavel-Chapelon F. (2013). Consumption of artificially and sugar-sweetened beverages and incident type 2 diabetes in the Etude Epidemiologique aupres des femmes de la Mutuelle Generale de l’Education Nationale-European Prospective Investigation into Cancer and Nutrition cohort. Am. J. Clin. Nutr..

[54] Greenwood D.C., Threapleton D.E., Evans C.E., Cleghorn C.L., Nykjaer C., Woodhead C., Burley V.J. (2014). Association between sugar-sweetened and artificially sweetened soft drinks and type 2 diabetes: systematic review and dose-response meta-analysis of prospective studies. Br. J. Nutr..

[55] Cozma A.I., Sievenpiper J.L., de Souza R.J., Chiavaroli L., Ha V., Wang D.D., Mirrahimi A., Yu M.E., Carleton A.J., Di Buono M., Jenkins A.L. (2012). Effect of fructose on glycemic control in diabetes: a systematic review and meta-analysis of controlled feeding trials. Diabetes Care.

[56] Sievenpiper J.L., Chiavaroli L., de Souza R.J., Mirrahimi A., Cozma A.I., Ha V., Wang D.D., Yu M.E., Carleton A.J., Beyene J. (2012). ‘Catalytic’ doses of fructose may benefit glycaemic control without harming cardiometabolic risk factors: A small meta-analysis of randomised controlled feeding trials. Br. J. Nutr..

[57] Livesey G., Taylor R. (2008). Fructose consumption and consequences for glycation, plasma triacylglycerol, and body weight: Meta-analyses and meta-regression models of intervention studies. Am. J. Clin. Nutr..

[58] Turner R.C., Millns H., Neil H.A., Stratton I.M., Manley S.E., Matthews D.R., Holman R.R. (1998). Risk factors for coronary artery disease in non-insulin dependent diabetes mellitus: United Kingdom Prospective Diabetes Study (UKPDS: 23). BMJ.

[59] Jeppesen J., Hein H.O., Suadicani P., Gyntelberg F. (1998). Triglyceride concentration and ischemic heart disease: An eight-year follow-up in the Copenhagen Male Study. Circulation.

[60] Topping D.L., Mayes P.A. (1972). The immediate effects of insulin and fructose on the metabolism of the perfused liver. Changes in lipoprotein secretion, fatty acid oxidation and esterification, lipogenesis and carbohydrate metabolism. Biochem. J..

[61] McDevitt R.M., Bott S.J., Harding M., Coward W.A., Bluck L.J., Prentice A.M. (2001). De novo lipogenesis during controlled overfeeding with sucrose or glucose in lean and obese women. Am. J. Clin. Nutr..

[62] Acheson K.J., Schutz Y., Bessard T., Anantharaman K., Flatt J.P., Jequier E. (1988). Glycogen storage capacity and de novo lipogenesis during massive carbohydrate overfeeding in man. AJCN.

[63] Chong M.F., Fielding B.A., Frayn K.N. (2007). Mechanisms for the acute effect of fructose on postprandial lipemia. Am. J. Clin. Nutr..

[64] Duffey K.J., Gordon-Larsen P., Steffen L.M., Jacobs D.R., Popkin B.M. (2010). Drinking caloric beverages increases the risk of adverse cardiometabolic outcomes in the Coronary Artery Risk Development in Young Adults (CARDIA) Study. Am. J. Clin. Nutr..

[65] Raben A., Moller B.K., Flint A., Vasilaris T.H., Christina Moller A., Juul Holst J., Astrup A. (2011). Increased postprandial glycaemia, insulinemia, and lipidemia after 10 weeks’ sucrose-rich diet compared to an artificially sweetened diet: A randomised controlled trial. Food Nutr. Res..

[66] Sobrecases H., Le K.A., Bortolotti M., Schneiter P., Ith M., Kreis R., Boesch C., Tappy L. (2010). Effects of short-term overfeeding with fructose, fat and fructose plus fat on plasma and hepatic lipids in healthy men. Diabetes Metab..

[67] Swarbrick M.M., Stanhope K.L., Elliott S.S., Graham J.L., Krauss R.M., Christiansen M.P., Griffen S.C., Keim N.L., Havel P.J. (2008). Consumption of fructose-sweetened beverages for 10 weeks increases postprandial triacylglycerol and apolipoprotein-B concentrations in overweight and obese women. Br. J. Nutr..

[68] Sievenpiper J.L., Carleton A.J., Chatha S., Jiang H.Y., de Souza R.J., Beyene J., Kendall C.W., Jenkins D.J. (2009). Heterogeneous effects of fructose on blood lipids in individuals with type 2 diabetes: Systematic review and meta-analysis of experimental trials in humans. Diabetes Care.

[69] Lecoultre V., Egli L., Carrel G., Theytaz F., Kreis R., Schneiter P., Boss A., Zwygart K., Lê K.A., Bortolotti M. (2013). Effects of fructose and glucose overfeeding on hepatic insulin sensitivity and intrahepatic lipids in healthy humans. Obesity.

[70] Theytaz F., Noguchi Y., Egli L., Campos V., Buehler T., Hodson L., Patterson B.W., Nishikata N., Kreis R., Mittendorfer B. (2012). Effects of supplementation with essential amino acids on intrahepatic lipid concentrations during fructose overfeeding in humans. Am. J. Clin. Nutr..

[71] Johnston R.D., Stephenson M.C., Crossland H., Cordon S.M., Palcidi E., Cox E.F., Taylor M.A., Aithal G.P., Macdonald I.A. (2013). No difference between high-fructose and high-glucose diets on liver triacylglycerol or biochemistry in healthy overweight men. Gastroenterology.

[72] Ouyang X., Cirillo P., Sautin Y., McCall S., Bruchette J.L., Diehl A.M., Johnson R.J., Abdelmalek M.F. (2008). Fructose consumption as a risk factor for non-alcoholic fatty liver disease. J. Hepatol..

[73] Kanerva N., Sandboge S., Kaartinen N.E., Mannisto S., Eriksson J.G. (2014). Higher fructose intake is inversely associated with risk of nonalcoholic fatty liver disease in older Finnish adults. Am. J. Clin. Nutr..

[74] Abdelmalek M.F., Suzuki A., Guy C., Unalp-Arida A., Colvin R., Johnson R.J., Diehl A.M., Nonalcoholic Steatohepatitis Clinical Research Network (2010). Increased fructose consumption is associated with fibrosis severity in patients with nonalcoholic fatty liver disease. Hepatology.

[75] Lee O., Bruce W.R., Dong Q., Bruce J., Mehta R., O’Brien P.J. (2009). Fructose and carbonyl metabolites as endogenous toxins. Chem. Biol. Interact..

[76] Wang X., Jia X., Chang T., Desai K., Wu L. (2008). Attenuation of hypertension development by scavenging methylglyoxal in fructose-treated rats. J. Hypertens..

[77] Johnson R.J., Nakagawa T., Sanchez-Lozada L.G., Shafiu M., Sundaram S., Le M., Ishimoto T., Sautin Y.Y., Lanaspa M.A. (2013). Sugar, uric acid, and the etiology of diabetes and obesity. Diabetes.

[78] Perez-Pozo S.E., Schold J., Nakagawa T., Sanchez-Lozada L.G., Johnson R.J., Lillo J.L. (2010). Excessive fructose intake induces the features of metabolic syndrome in healthy adult men: Role of uric acid in the hypertensive response. Int. J. Obes..

[79] Choi H.K., Curhan G. (2008). Soft drinks, fructose consumption, and the risk of gout in men: Prospective cohort study. BMJ.

[80] Choi H.K., Willett W., Curhan G. (2010). Fructose-rich beverages and risk of gout in women. JAMA.

[81] Cox C.L., Stanhope K.L., Schwarz J.M., Graham J.L., Hatcher B., Griffen S.C., Bremer A.A., Berglund L., McGahan J.P., Keim N.L. (2012). Consumption of fructose- but not glucose-sweetened beverages for 10 weeks increases circulating concentrations of uric acid, retinol binding protein-4, and gamma-glutamyl transferase activity in overweight/obese humans. Nutr. Metab..

[82] Wang D.D., Sievenpiper J.L., de Souza R.J., Chiavaroli L., Ha V., Cozma A.I., Mirrahimi A., Yu M.E., Carleton A.J., Di Buono M. (2012). The effects of fructose intake on serum uric acid vary among controlled dietary trials. J. Nutr..

[83] Ruanpeng D., Thongprayoon C., Cheungpasitporn W., Harindhanavudhi T. (2017). Sugar and artificially sweetened beverages linked to obesity: A systematic review and meta-analysis. QJM.

[84] Cheungpasitporn W., Thongprayoon C., Edmonds P.J., Srivali N., Ungprasert P., Kittanamongkolchai W., Erickson S.B. (2015). Sugar and artificially sweetened soda consumption linked to hypertension: A systematic review and meta-analysis. Clin. Exp. Hypertens..

[85] Jayalath V.H., de Souza R.J., Ha V., Mirrahimi A., Blanco-Mejia S., Di Buono M., Jenkins A.L., Leiter L.A., Wolever T.M., Beyene J. (2015). Sugar-sweetened beverage consumption and incident hypertension: A systematic review and meta-analysis of prospective cohorts. Am. J. Clin. Nutr..

[86] Xi B., Huang Y., Reilly K.H., Li S., Zheng R., Barrio-Lopez M.T., Martinez-Gonzalez M.A., Zhou D. (2015). Sugar-sweetened beverages and risk of hypertension and CVD: A dose-response meta-analysis. Br. J. Nutr..

[87] Huang C., Huang J., Tian Y., Yang X., Gu D. (2014). Sugar sweetened beverages consumption and risk of coronary heart disease: A meta-analysis of prospective studies. Atherosclerosis.

[88] Narain A., Kwok C.S., Mamas M.A. (2016). Soft drinks and sweetened beverages and the risk of cardiovascular disease and mortality: A systematic review and meta-analysis. Int. J. Clin. Pract..

[89] Imamura F., O’Connor L., Ye Z., Mursu J., Hayashino Y., Bhupathiraju S.N., Forouhi N.G. (2015). Consumption of sugar sweetened beverages, artificially sweetened beverages, and fruit juice and incidence of type 2 diabetes: Systematic review, meta-analysis, and estimation of population attributable fraction. BMJ.

[90] Xi B., Li S., Liu Z., Tian H., Yin X., Huai P., Tang W., Zhou D., Steffen L.M. (2014). Intake of fruit juice and incidence of type 2 diabetes: A systematic review and meta-analysis. PLoS ONE.

[91] Wijarnpreecha K., Thongprayoon C., Edmonds P.J., Cheungpasitporn W. (2016). Associations of sugar- and artificially sweetened soda with nonalcoholic fatty liver disease: A systematic review and meta-analysis. QJM.

[92] Chiavaroli L., de Souza R.J., Ha V., Cozma A.I., Mirrahimi A., Wang D.D., Yu M., Carleton A.J., Di Buono M., Jenkins A.L. (2015). Effect of Fructose on Established Lipid Targets: A Systematic Review and Meta-Analysis of Controlled Feeding Trials. J. Am. Heart Assoc..

[93] Jamnik J., Rehman S., Blanco Mejia S., de Souza R.J., Khan T.A., Leiter L.A., Wolever T.M., Kendall C.W., Jenkins D.J., Sievenpiper J.L. (2016). Fructose intake and risk of gout and hyperuricemia: A systematic review and meta-analysis of prospective cohort studies. BMJ Open.

[94] WHO (2015). Guideline: Sugars Intake for Adults and Children.

[95] SACN Carbohydrate and Health: A Scientific Advisory Committee on Nutrition Report; 2015. https://www.gov.uk/government/publications/sacn-carbohydrates-and-health-report.

[96] Department of Health and Human Services and U.S.A. Department of Agriculture *2015–2020 Dietary Guidelines for Americans*, 8th Ed; December 2015. https://health.gov/dietaryguidelines/2015/guidelines/.

[97] Erickson J., Slavin J. (2015). Total, added, and free sugars: Are restrictive guidelines science-based or achievable?. Nutrients.

[98] ANSES Avis de l’ANSES relatif à l’établissement de recommandations d’apports en sucres; Jan 2017. https://www.anses.fr/fr/system/files/NUT2012SA0186Ra.pdf.

