# Supplementary material for: French Recommendations for Sugar Intake in Adults: A Novel Approach Chosen by ANSES
Source: Nutrients. 2018 Jul 29;10(8):989. doi: 10.3390/nu10080989 (PMC6115815; doi:10.3390/nu10080989)
Supplement: Supplementary file 1 [file nutrients-10-00989-s001.pdf]

Table S1: **<u>Summary of findings</u>**

Effects of sugars on body weight control

- Sugars do not decrease energy expenditure.
- Studies having evaluated the effects of sugars on food intake do not allow to reach conclusions at this time.
- Sugar supplementation, with dietary intake otherwise left ad-libitum, is associated with body weight gain.
- Epidemiological studies show an association between sugar consumption and body weight gain.

Effects of sugars on glucose homeostasis

- High fructose intake (> 80 g/d) is associated with hepatic insulin resistance without hyperglycemia.
- High fructose or sucrose intake is not associated with impaired insulin-mediated glucose disposal independently of changes in body weight.

Effects of sugars on blood lipids and cardiovascular risk

- There is no association between sugar intake and LDL- or HDL-cholesterol.
- There is an association between sugar intake and fasting and postprandial blood triglyceride concentration.
- The minimal daily fructose dose associated with increased postprandial triglyceride concentration is estimated to be 50 g/day.

Effects of sugar on intrahepatic fat

- A very high (> 150g/day) fructose intake can increase intrahepatic fat concentration,
- The scientific data available at the time of assessment is not sufficient to assess the association between sugar intake and NAFLD.

Effects of sugars on blood uric acid concentration

- Changes in sugar intake are not associated with changes in uric acid concentration, unless they involve very large amount of fructose (ca 200g/day) together with an excess total energy intake.
- Consumption of sugar-sweetened beverages (SSBs) is associated with the development of gout.
